# Exploring Titanium(IV) Complexes as Potential Antimicrobial Compounds

**DOI:** 10.3390/antibiotics11020158

**Published:** 2022-01-26

**Authors:** Israel Rodríguez, Lauren Fernández-Vega, Andrea N. Maser-Figueroa, Branlee Sang, Patricia González-Pagán, Arthur D. Tinoco

**Affiliations:** Department of Chemistry, University of Puerto Rico-Río Piedras Campus, San Juan 00925, Puerto Rico; israel.rodriguez6@upr.edu (I.R.); lauren.fernandez@upr.edu (L.F.-V.); andrea.maser@upr.edu (A.N.M.-F.); branlee.sang@upr.edu (B.S.); patricia.gonzalez6@upr.edu (P.G.-P.)

**Keywords:** transmetalation, titanium(IV) antimicrobial compounds, iron chelation

## Abstract

Due to the rapid mutation of pathogenic microorganisms, drug-resistant superbugs have evolved. Antimicrobial-resistant germs may share their resistance genes with other germs, making them untreatable. The search for more combative antibiotic compounds has led researchers to explore metal-based strategies centered on perturbing the bioavailability of essential metals in microbes and examining the therapeutic potential of metal complexes. Given the limited knowledge on the application of titanium(IV), in this work, eight Ti(IV) complexes and some of their corresponding ligands were screened by the Community for Open Antimicrobial Drug Discovery for antimicrobial activity. The compounds were selected for evaluation because of their low cytotoxic/antiproliferative behavior against a human non-cancer cell line. At pH 7.4, these compounds vary in terms of their solution stability and ligand exchange lability; therefore, an assessment of their solution behavior provides some insight regarding the importance of the identity of the metal compound to the antimicrobial therapeutic potential. Only one compound, Ti(deferasirox)_2_, exhibited promising inhibitory activity against the Gram-positive bacteria methicillin-resistant *Staphylococcus aureus* and minimal toxicity against human cells. The ability of this compound to undergo transmetalation with labile Fe(III) sources and, as a consequence, inhibit Fe bioavailability and ribonucleotide reductase is evaluated as a possible mechanism for its antibiotic effect.

## 1. Introduction

The urgent need for new classes of antimicrobial drugs to fight resistance and the evolution of superbugs has forced researchers to explore different strategies. The classical approach has been the synthesis of derivatives of already known compounds that can specifically attack microbes rather than human cells, the majority of which are purely organic compounds. This approach is quickly reaching the limit of its possibilities. Given the central role that essential metals can play in microbial growth and the distinctive chemistry that can be facilitated by metals coupled with the varied structures and properties that they can adopt from ligand complexation, metal-centered antimicrobial strategies are an exciting new research avenue. One approach is developing compounds that can uniquely perturb the essential metal homeostasis within pathogenic microbes and possibly even attenuate their bioavailability [1,2,3,4]. Another approach is assessing the therapeutic potential of metals in ion or complex form. Bismuth(III) and silver(I) are currently the only metals used in the treatment of certain infections [5,6,7,8,9]. Ag(I) has been tested in combination with different classes of antibiotic agents and exhibits excellent synergism [10]. The Ag(I) complex of penicillin-G expands the spectrum of the effect of the antibiotic so that it might be useful against Gram-negative bacteria [11]. A variety of organometallic derivatives of known antibacterial drugs have been explored [12]. In this regard, the ferrocenyl moiety holds tremendous potential as a multipurpose antimicrobial agent [13]. A recent review highlights some of the most important antibiotic metal complexes developed over the last decade [14].

Recently Blaskovich et al. undertook a very ambitious effort to screen the results of over 295,000 compounds that were submitted to the Community for Open Antimicrobial Drug Discovery (CO-ADD) from laboratories throughout the world with the intent of identifying promising antimicrobial metal-based compounds with little to no toxicity against human cells [9]. The CO-ADD provides a free service to test compounds at a one dose concentration (32 μg/mL) against four Gram-negative bacteria (*Escherichia coli*, *E. coli*; multidrug-resistant *Klebsiella pneumoniae*, *K. pneumoniae* (MDR); *Acinetobacter baumannii*, *A. baumannii*; and *Pseudomonas aeruginosa*; *P. aeruginosa*), the Gram-positive bacteria (methicillin-resistant *Staphylococcus aureus,* methicillin resistant *S. aureus* (MRSA)), and the fungi (*Candida albicans*, *C. albicans* and *Cryptococcus neoformans*, *C. neoformans*) [15]. Depending on the potency demonstrated, the compounds are then evaluated in a dose–response screening (32–0.25 μg/mL) against the microbes and are also tested for cytotoxicity against the human embryonic kidney cell line HEK293 and hemolysis against human red blood cells. Blaskovich et al. found that 906 metal-based compounds had been screened, of which 88 met the criteria of displaying a minimum inhibitory concentration (MIC) of ≤16 μg/mL against at least one microbe and no toxicity against the human cells [9]. They made a valiant call to the academic community to continue the effort to discover new antimicrobial metal-based compounds and contribute compounds from their own labs to the CO-ADD. To our surprise, not one of the 906 metal-based compounds in the study of Blaskovich et al. was a Ti(IV) compound [9]. In this work, we respond to this call by contributing a series of titanium(IV) compounds.

Titanium, the ninth most abundant element on the earth’s crust, is generally non-toxic to the human body and is commonly used in the medical field in prosthetics [16,17,18]. Depending on how Ti(IV) is coordinated by ligands in coordination compounds, the metal ion can be transformed into a cytotoxic species, which is why it has been explored for anticancer applications [17,19,20,21]. Two Ti(IV) compounds, titanocene dichloride and budotitane, were tested in clinical trials as anticancer drug candidates, although they did not advance to the drug market [22,23]. Ti(IV) has been far less investigated for its antimicrobial properties. Due to their semiconductor properties, TiO_2_ nanoparticles (TiO_2_ NPs) in the anatase and rutile forms have been extensively studied for their UV-induced photocatalytic ability to generate reactive oxygen species, which can effectively kill microbes [24]. TiO_2_ NPs have been shown to kill Gram-positive and Gram-negative bacteria [25,26,27] and can be useful in the disinfection of water contaminated with *E. coli* [28]. TiO_2_ NPs also demonstrate some antifungal capacity against a variety of fungi [29,30,31]. In *C. albicans*, the NPs cause morphological changes that indicate that their direct contact with the cells is the main mechanism of cell death [30]. Much less is known about the microbial properties of soluble Ti(IV) compounds. The German physician Julius Pick observed that hydrolyzed Ti(IV) sulfate and Ti(IV) mono- and disalicylates could inhibit putrefaction of protein-rich materials and served as an effective topical and oral treatment of tuberculosis [27]. These compounds were likely some form of titanyl (TiO^2+^) species in solution [17]. McCue et al. reported that titanyl sulfate could inhibit the growth of *E. coli*, *S. typhimurium*, and *P. aeruginosa*, possibly by a general inhibition of the bacterial serine proteases [32]. The bacteria exhibited loss of motility and compromised metabolism. Motility is linked to bacterial virulence. Titanyl sulfate was also able to inhibit *E. coli* in soil, but this ability was decreased in the presence of increasing amounts of oxalate, possibly owed to the formation of an inactive titanyl oxalate species [33,34]. In related studies, white mice fed titanyl oxalate supplied in bread exhibited higher weight in the form of lean mass compared to control mice [33,35], which might have been correlated with a 50% decrease in the total intestinal bacterial count [35]. The bacteriostatic/bactericidal activity of titanyl oxalate in these mice is probably attributable to the acidification and dissociation of the compound during digestion, liberating the titanyl moiety [34].

**Figure 1:**
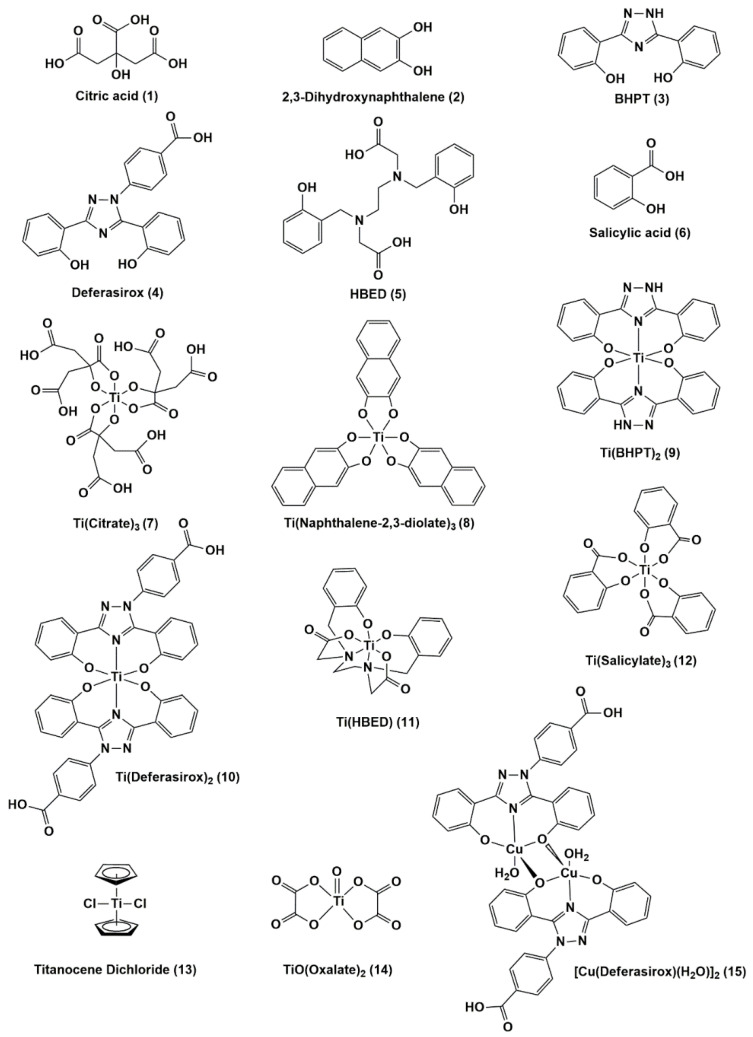
The known/proposed structures of eight Ti(IV) complexes and some of their corresponding ligands and the compound [Cu(Deferasirox)(H_2_O)]_2_ that were tested for antimicrobial activity.

With the context of what is known about the antimicrobial behavior of soluble Ti(IV) compounds, we selected eight Ti(IV) compounds that are commonly utilized/studied in our laboratory to be screened by the CO-ADD (Figure 1). These compounds were selected based on our own in-house assessment of their low antiproliferative activity/cytotoxicity against the noncancerous lung cell line MRC-5 and because they range in their aqueous solution stability and ligand exchange lability. The results reveal little to no activity against the screened microbes with the exception of one compound (titanium(IV) bis(deferasirox) against MRSA. Insight into the inhibitory mechanism of this compound is explored with regard to its iron-binding capability.

## 2. Materials and Methods

### 2.1. Chemicals

*N*,*N*′-di(o-hydroxybenzyl)ethylenediamine-*N*,*N*′-diacetic acid monohydrochloride hydrate (HBED·HCl·H_2_O) was purchased from Strem Chemicals, Inc. (Newburyport, MA, USA). 4-[(3Z,5E)-3,5-Bis(6-oxocyclohexa-2,4-dien-1-ylidene)-1,2,4-triazolidin-1-yl] benzoic acid (deferasirox) was purchased from Focus Synthesis LCC (San Diego, CA, USA). 2-[3-(2-Hydroxyphenyl)-1H-1,2,4-triazol-5-yl]phenol (BHPT) was purchased from Princeton Biomolecular Research, Inc. (Sonesta ES Suites South Brunswick-Princeton, NJ, USA). Trisodium citrate (Na_3_Citrate) was purchased from Thermo Fisher Scientific (Waltham, MA, USA). 2,3-dihydroxynaphthalene, salicylic acid, titanium(IV) tetrachloride (TiCl_4_), sodium chloride, sodium hydroxide pellets, hydrochloric acid (37%), and ethanol (99.5%, 200 proof) were obtained from Fisher Scientific (Nazareth, PA, USA). Cisplatin was purchased from Abcam (Cambridge, UK). *N*,*N*-dimethylformamide (DMF, 99.9%, HPLC grade) and dimethyl sulfoxide (DMSO, 99.9%) were purchased from Sigma-Aldrich (St. Louis, MN, USA). Compressed Argon (Ar, UN1006) was obtained from Messer (Puerto Rico, USA). MRC-5 human lung normal cells were obtained from ATCC (CCL-185 and CCL-171, respectively) (Manassas, VA, USA). Mycoplasm removal agent was used in the preparation of cell stocks (Bio-Rad Laboratories, Inc., Hercules, CA, USA). The cell line was cultured in phenol red Dulbecco’s Modified Eagle’s Medium (DMEM, D6429) Millipore Sigma, Burlington, MA, USA) supplemented with 10% Fetal Bovine Serum (FBS; HyClone, Thermo Fisher Scientific, Waltham, MA, USA) and 1% of penicillin-streptomycin (Prepared from the solid obtained from Sigma-Aldrich (St. Louis, MO, USA) and prepared as follows: Streptomycin 11 mg/mL and Penicillin 7 mg/mL) at 37 °C in a humidified atmosphere of 5% (*v*/*v*) CO_2_. Phenol red-free DMEM (CellGro REF 17-205-CV, Thermo Fisher Scientific, Waltham, MA, USA) supplemented with L-glutamine (Millipore Sigma, Burlingtong, MA, USA) at 2.4 mM, 10% FBS, and 1% of penicillin-streptomycin was used for cell viability studies. 3-(4,5-dimethylthiazole-2-yl)-2,5-diphenyl tetrazolium bromide (MTT) was purchased from Sigma-Aldrich (St. Louis, MO, USA). Autoclavable 0.2 micron filters were obtained from Thermo Fisher Scientific (Waltham, MA, USA) and were used to sterile filter solutions. Tris (biotechnology grade) from Amresco—VWR International LLC (Solon, OH, USA) was used to prepare 1M Tris buffer (pH 11). Dodecyl sulfate sodium salt (SDS, electrophoresis grade, 98% pure) was obtained from Acros Organics—Thermo Fisher Scientific (Waltham, MA, USA). Trypan blue solution (0.4%) was purchased from Sigma-Aldrich (St. Louis, MO, USA) and was used as a cell staining solution. Ingredients to prepare sterile 10X Phosphate buffer saline (PBS) was obtained from Thermo Fisher Scientific (Waltham, MA, USA) and was diluted tenfold to produce 1X PBS (pH 7.4). A Hausser Scientific hemacytometer (Thermo Fisher Scientific (Waltham, MA, USA)) was used to perform cell counts. All commercially purchased materials were of high purity and used as received. All aqueous solutions were prepared with autoclaved (121 °C and 18 psi) high-quality nanopure water (18.2 MΩ·cm resistivity) at 25 °C, PURELAB flex system (ELGA Corporation, Woodridge, IL, USA).

### 2.2. Sources of Metal Complexes

The metal compounds submitted to the CO-ADD for screening were either commercially purchased or synthesized and confirmed to be at ≥95% purity. Bis(cyclopentadienyl)titanium(IV) dichloride (aka titanocene dichloride; 248.96 g/mol) was purchased from Sigma Aldrich. Potassium dioxalatoxotitanate(IV) dihydrate (K_2_[TiO(Oxalate)_2_]·2H_2_O; 354.13 g/mol) was obtained from Fisher Scientific. Ti(HBED)·0.5H_2_O (441.40 g/mol), Ti(Deferasirox)_2_·H_2_O (790.57 g/mol), Ti(BHPT)_2_·2H_2_O·C_3_H_6_O (C_3_H_6_O = acetone) (644.46 g/mol), and K_2_[Ti(Naphthalene-2,3-diolate)_3_]·3H_2_O (654.58 g/mol) were synthesized by following our previously published protocols [36,37,38]. Na_2_[Ti(Salicylate)_3_]·4H_2_O·0.5C_2_H_6_O (C_2_H_6_O = ethanol) (597.26 g/mol) and K_2_[Ti(Citrate)_3_]·2H_2_O (732.41 g/mol) were synthesized using published synthetic routes [39,40]. [Cu(Deferasirox)H_2_O]_2_ (905.83 g/mol) was synthesized following our laboratory protocol [41]. The molecular formulas for all synthesized metal compounds submitted to the CO-ADD screening were proposed by using C,H,N elemental analyses performed by Atlantic Microlab, Inc. (Norcross, GA, USA) and are within 0.4% of the calculated values for the proposed formula and where applicable, these formulas are consistent with our previously published use of the compounds [36,37,38,42,43]. For simplicity, all compounds in this study will be referred to without their solvates or co-crystals.

### 2.3. Instrumentation

Absorbance measurements were conducted on the Agilent Technologies Cary Series UV-Vis spectrophotometer Cary 100 UV-Vis model using the UV Cary Scan software version v.20.0.470. All analyses are carried out with a scan of 600–200 nm. pH titrations were conducted with Thermoscientific Orion Star A211 and an Orion 9157BNMD electrode, calibrated at pH 4, 7, and 10 with standard buffer solutions. X-ray diffraction was performed using a Rigaku XtalLAB SuperNova single-micro-focus copper-K5-007 radiation (5-007 = 1.5417 Å) source equipped with a HyPix3000 X-ray detector in 50 kV transmission mode and 1 mA in CrystAllisPRO software ver. 1.171.39.43c. A Nicolet iS50 FTIR Spectrometer (Thermo Fisher Scientific, Waltham, MA, USA) was used to collect FTIR absorbance spectra. The EDS was obtained with 20 kV operating voltage in a scanning electron microscope JEOL JSM-5800LV. An Infinite M200 PRO Tecan Microplate Reader was used for cell viability measurements.

### 2.4. MRC-5 Cell Viability Study of Dose–Response Treatment with Different Compounds by the MTT Assay

A thawed stock of MRC-5 cells was washed with 1X PBS and resuspended in phenol red DMEM media (supplemented with 10% FBS and 1% of penicillin-streptomycin) and then seeded in a 100 mm × 20 mm (complete, O.D. x H) petri dish and grown in a 5% (*v*/*v*) CO_2_ humidified atmosphere at 37 °C. At least three passages were performed to ensure the integrity of the cells. After this point, cells were collected, thoroughly washed with 1X PBS, and then resuspended in phenol red-free DMEM (supplemented with 10% FBS, 1% Streptomycin/Penicillin, and 2.4 mM L-glutamine) at a 2.0 × 10^5^ cells/mL concentration. Approximately 100 µL of the cell solution was seeded into wells of several 96-well plates to begin the MTT assay [44], which was performed in the 5% (*v*/*v*) CO_2_ humidified atmosphere at 37 °C. At least one lane of wells in each plate was supplied media without cells as a control. After the plates were left incubating for one day, cells were treated with 100 µL of 1 to 100 μM final concentrations of the following compounds: K_2_[Ti(Naphthalene-2,3-diolate)_3_], titanocene dichloride, K_2_[TiO(Oxalate)_2_], K_2_[Ti(Citrate)_3_], Na_2_[Ti(Salicylate)_3_], [Cu(Deferasirox)(H_2_O)]_2_, 2,3-dihydroxynaphthalene, salicylic acid, and Na_3_Citrate. Most of the compounds were dissolved in 1X PBS buffer at pH 7.4. Due to poor aqueous solubility, [Cu(Deferasirox)(H_2_O)]_2_ was extensively sonicated in the buffer for 30 min at 30 °C until no particulate matter could be observed. Titanocene dichloride was prepared as a fresh stock solution in DMSO and then diluted in 0.1 M NaCl (aq) instead of 1X PBS (to minimize hydrolysis induced precipitation [45]) to the desired concentration while maintaining a constant 1% (*v*/*v*) DMSO background (0.5% DMSO after mixing with the well media). A control of cells treated with media containing 1% (*v*/*v*) DMSO was included in the study. The titanocene solutions were used within an hour of preparation in the final cell solution. Cisplatin at 40 μM final concentration after mixing with cells was used as a negative control. The cisplatin stock solution had to be prepared in DMF (and not DMSO [46]), then diluted in buffer and used soon after dilution. At 3 h before a total of 72 h of incubation with the compounds, the cells were treated with 25 µL of 9 mg/mL MTT prepared in 1X PBS and sterile-filtered. Upon completion of the three hours, 50 µL of 10% (*w*/*v*) SDS (prepared in 0.1 M Tris buffer at pH 11) [47] was added to solubilize the purple formazan product crystals and left overnight in the incubator. Control wells in the plates consisted of: (1) Untreated cells + MTT as the measure of 100% viable cell growth in the media and (2) Media with no cells + MTT as the measure of zero cells in the media. Two sets of compounds could be tested per plate. The plates were read at the wavelength of 570 nm (absorbance of the formazan product at pH 10.0) and 800 nm (background control) using a Tecan plate reader. The absorbance of all wells was compared to the absorbance of the untreated cells with the MTT set as the 100% viable cell standard and the absorbance of the zero cell control set as the 0% viable cell standard. The 40 μM cisplatin negative control produced 5.2 ± 1.4% viable cells in accordance with expectation. Nonlinear regression in Origin 8.5 was utilized to fit the growth curves using the pharmacology dose–response equation to determine the half-maximal inhibitory concentration (IC_50_) and the corresponding standard deviation. All samples were tested six times.

### 2.5. Solid-State and pH and Concentration Dependent Characterization of Ti(IV) Salicylate Complexation

The complex Na_2_[Ti(salicylate)_3_] has been previously characterized by single-crystal X-ray diffraction [39]. In this work, the compound was evaluated by powder X-ray diffraction (PXRD), Fourier transform infrared (FT-IR) spectroscopy, and energy-dispersive X-ray spectroscopy (EDS).

The Ti(IV) and salicylic acid solution complexation at a mole equivalent ratio of 1:3 were studied under pH-dependent conditions by UV-Vis absorbance spectroscopy (250 to 600 nm) at 25.0 °C. Stock solutions of salicylic acid and TiCl_4_ were prepared in pure ethanol. The TiCl_4_ stock solution preparation required that the ethanol be deoxygenated by purging with Ar gas and chilled in an ice-water bath prior to syringe addition of TiCl_4_. Once prepared, the solution could be open to the air. The Ti(IV) concentration of this solution was determined by our previously published 2,3-dihydroxynaphthalene colorimetric assay [48]. The ligand was diluted to 150 μM to a final background of 50:50 ethanol:water containing 0.1 M NaCl (to maintain an ionic strength (I) of 0.1 M) and then divided into two batches. One batch was pH titrated with aliquots of either 0.1 M or 1.0 M HCl by micropipette to acidify the solution. The other batch was pH titrated with aliquots of either 0.1 M or 1.0 M NaOH to basify the solution. The selection of acid or base concentration was to maintain the total volume of addition low to not alter the concentration of the analyte by greater than 5%. After the addition of each aliquot of acid or base, the solutions were equilibrated for at least 10 min, when the millivoltage reading remained constant. At predefined pH values, 1 mL of the titration solution was removed for analysis by UV-Vis absorbance. The changes in concentration were accounted for. The same pH titration was performed for the ligand (150 μM) in the presence of Ti(IV) (50 μM). In total, the pH-metric titrations of the ligand in the presence and absence of Ti(IV) were performed in the pH range 2.0–12.0.

A concentration-dependent study was performed. A stock 10 mM Na_2_[Ti(Salicylate)_3_] solution was prepared in DMF because it is not soluble in ethanol and has limited solubility in water. The solution was diluted to the concentrations 5, 1, 0.5, 0.25, and 0.15 mM with a fixed background of 50:50 DMF:water, containing 0.1 M NaCl. The solutions were left to equilibrate overnight and were all pH ~ 4.7 without any pH adjustment. UV-Vis absorbance spectra of the solutions were collected.

### 2.6. Compound Solution Preparation by the CO-ADD

Dry compounds ranging from 1–2 mg were weighed into properly labeled Eppendorf tubes and sealed with parafilm. The compounds were used as received by the CO-ADD. For the screenings, most compounds were dissolved to a stock concentration of 10 mg/mL in DMSO. K_2_[Ti(Citrate)_3_], Na_3_Citrate, and K_2_[TiO(Oxalate)_2_] were dissolved in warm water. [Cu(Deferasirox)(H_2_O)]_2_ was dissolved in pyridine after its solubility was determined to be excellent in pyridine due to coordination of the solvent to the metal producing [Cu(Deferasirox)(pyridine)]_2_ in situ [41]. Samples were diluted in water to a final testing concentration of 32 µg/mL while keeping the final co-solvent concentration to a maximum of 0.5%. Where applicable, compounds were serially diluted 1:2 fold eight times. Each sample concentration was prepared in 384-well plates, non-binding surface plate (NBS; Corning 3640) for each bacterial/fungal strain, tissue-culture treated (TC-treated; Corning 3712/3764), black for mammalian cell types and polypropylene 384-well (PP; Corning 3657) for hemolysis assays, all in duplicate (*n* = 2), and keeping the final DMSO concentration to a maximum of 0.5%. All sample preparation was conducted using liquid handling robots.

### 2.7. CO-ADD Antibacterial Assays

For the bacterial assays, four Gram-negative bacteria (*E. coli* (ATCC 25922, Manassas, VA, USA); *K. pneumoniae* (ATCC 700603), *A. baumannii*; (ATCC 19606), and *P. aeruginosa* (ATCC 27853) and the Gram-positive bacteria (*S. aureus* MRSA (ATCC 43300) were used. Each strain was cultured in cation-adjusted Mueller Hinton broth (CAMHB; Bacto Laboratories 212322, Mount Pritchard, Australia) at 37 °C overnight. A sample of each culture was then diluted 40-fold in fresh CAMHB and incubated at 37 °C for 1.5–3 h. The resultant mid-log phase cultures were diluted with CAMHB (CFU/mL measured by OD_600_), then added to each well of the compound-containing plates (384-well non-binding surface (NBS) plates; Corning CLS3640, Millipore Sigma, Burlington, MA, USA), giving a cell density of 5 × 10^5^ CFU/mL and a total volume of 50 µL. Plates were covered and incubated at 37 °C for 18 h without shaking. Inhibition of bacterial growth was determined by measuring absorbance at 600 nm (OD_600_) with a Tecan M1000 Pro monochromator plate reader, using media-only as negative control and bacteria without inhibitors as a positive control. Minimum inhibitory concentration (MIC) values were determined as the lowest concentration at which the growth was inhibited at greater than or equal to 75%. Colistin sulfate (Sigma C4461) and vancomycin HCl (Sigma 861987) were used as internal controls on each plate for Gram-negative and Gram-positive bacteria, respectively. These inhibitors exhibited MIC values (μg/mL) against the microbes within the expected range.

### 2.8. CO-ADD Antifungal Assays

For the fungal assays, the fungi (*C. albicans* (ATCC 90028) and *C. neoformans*) (ATCC 208821) were used. Both fungi (yeast) strains were cultured for 3 days on Yeast Extract-Peptone Dextrose (YPD; Becton Dickinson 242720, Thermo Fisher Scientific, Waltham, MA, USA) agar at 30 °C. A yeast suspension of 1 × 10^6^ to 5 × 10^6^ CFU/mL (as determined by OD_530_) was prepared from five colonies from the agar plates, subsequently diluted with Yeast Nitrogen Base media (YNB; Becton Dickinson 233520, Thermo Fisher Scientific, Waltham, MA, USA), and added to each well of the compound-containing plates (384-well plates, NBS; Corning CLS3640) giving a final cell density of 2.5 × 10^3^ CFU/mL and a total volume of 50 μL. Plates were covered and incubated at 35 °C for 36 h without shaking. Growth inhibition of *C. albicans* was determined by measuring absorbance at 630 nm (OD_630_), while the growth inhibition of *C. neoformans* was determined by measuring the difference in absorbance between 600 and 570 nm (OD_600–570_), after the addition of resazurin (0.001% final concentration; Sigma R7017) and incubation at 35 °C for 2 h, using media-only as a negative control and fungi without inhibitors as a positive control. MIC values were determined as the lowest concentration at which the growth was inhibited at greater than or equal to 75%. Fluconazole (Sigma F8929) was used as an internal control on each plate for both strains. These inhibitors exhibited MIC values (μg/mL) against the microbes within the expected range.

### 2.9. CO-ADD Cytotoxicity Assays

HEK293 ATCC CRL-1573 human embryonic kidney cells were counted manually in a Neubauer hemocytometer and added to compound-containing plates (384-well plates, tissue culture-treated (TC); Corning CLS3712), giving a final density of 5000 cells per well in a total volume of 50 µL, using Dulbecco’s Modified Eagle Medium (DMEM; Life Technologies 11995-073) with 10% FBS (GE SH30084.03). The cells were incubated together with the compounds for 20 h at 37 °C in 5% CO_2_. Cytotoxicity (or cell viability) was measured by fluorescence, ex: 560/10 nm, em: 590/10 nm (F_560/590_), after addition of 5 µL of 25 µg/mL resazurin (2.3 µg/mL final concentration; Sigma R7017) and after further incubation for 3 h at 37 °C in 5% CO_2_, using media-only as a negative control and cells without inhibitors as a positive control. CC_50_ (concentration at 50% cytotoxicity) were calculated by curve-fitting the inhibition values vs. log(concentration) using a sigmoidal dose–response function, with variable fitting values for the bottom, top, and slope. Tamoxifen (Sigma T5648) was used as an internal control on each plate and exhibited a CC_50_ value within the expected range.

### 2.10. CO-ADD Hemolysis Assays

Human whole blood (Australian Red Cross) was washed three times with three volumes of 0.9% NaCl and resuspended in a concentration of 0.5 × 10^8^ cells per mL, determined by manual cell count in a Neubauer hemocytometer. Washed cells were added to the compound-containing plates (384-well polypropylene plates (PP); Corning 3657) for a final volume of 50 μL, shaken and incubated for 1 h at 37 °C. After incubation, the plates were centrifuged at 1000× *g* for 10 min to pellet cells and debris, 25 µL of the supernatant was then transferred to reading plates (384 well, polystyrene plated (PS), Corning CLS3680), with hemolysis determined by measuring the supernatant absorbance at 405 mm (OD405), using cells without inhibitors as a negative control and cells with 1% Triton X-100 (Sigma T8787) as a positive control. HC_10_ and HC_50_ (concentration at 10% and 50% hemolysis, respectively) were calculated by curve fitting the inhibition values vs. log(concentration) using a sigmoidal dose–response function with variable fitting values for the top, bottom, and slope. Melittin (Sigma M2272) was used as an internal control on each plate and exhibited HC_10_ and HC_50_ values within the expected range.

## 3. Results

### 3.1. MRC-5 Cell Viability Study of Dose–Response Treatment with Different Compounds by the MTT Assay

**Figure 2:**
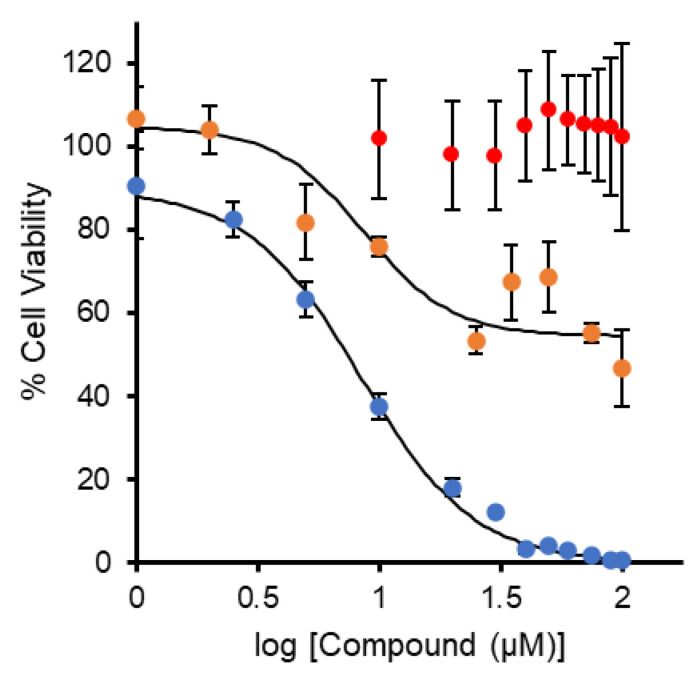
Dose–response treatment (*n* = 6) of MRC-5 cells with [Ti(salicylate)_3_]^2−^ (orange), [Cu(Deferasirox)(H_2_O)]_2_ (blue), and salicylic acid (red) at pH 7.4. The relative half-maximal inhibitory concentration was fit by nonlinear regression.

Ti(IV) compounds (Figure 1) selected for submission to the CO-ADD antimicrobial screening had to meet the criteria of low antiproliferative activity/cytotoxicity against a non-cancer human cell line as measured by the MTT assay at pH 7.4. For this purpose, we selected the MRC-5 lung cell line commonly used in our laboratory as a control in our cancer-related research and opted not to include a cancer cell line given the selectivity toward cancer cells that some of the compounds have demonstrated. We defined low antiproliferative activity/cytotoxicity as either a compound that exhibits an absolute IC_50_ value greater than 20 μM or inhibits cell proliferation at no greater than 50% within the micromolar concentration range. Absolute IC_50_ values are determined for compounds that inhibit cell proliferation at nearly 100%, whereas relative IC_50_ values are determined for inhibitory activity significantly less than 100%. Some of the IC_50_ values were previously determined, while others were measured in this work (Figure 2 and Table 1). All of the Ti(IV) compounds and the ligands met the criteria for low antiproliferative activity/cytotoxicity, while the compound [Cu(Deferasirox)(H_2_O)]_2_ did not. The data for the compounds that do not show any antiproliferative behavior are not shown with the exception of salicylic acid (monoanionic at pH 7.4), which is included as a representative example (Figure 2). As noted in a previous publication, deferasirox causes a nearly three-fold increase in the proliferation of MRC-5 cells for reasons that are not known [36].

### 3.2. Assesment of the Solution Behavior of the Ti(IV) Compounds

The Ti(IV) compounds in this study range widely in their aqueous solution stability and ligand exchange lability, especially at pH 7.4. Titanocene dichloride is extremely hydrolysis prone and undergoes virtually complete ligand dissociation at pH 7.4 and extensively hydrolyzed Ti(IV) rapidly precipitates from solution in the absence of a suitable chelator [45]. The complex Ti(Citrate)_3_ complex is ligand exchange labile [42,43]. In the absence of excess ligand (≥3:1 ligand:metal ratio), the Ti(IV) citrate speciation consists of a likely mixture of monocitrate, dicitrate, and tricitrate monoTi(IV) species, which nonetheless maintain the Ti(IV) ion in solution [50,51,52,53]. In the presence of excess ligand, the species Ti(Citrate)_3_^8−^ dominates [50,51,53]. The TiO(Oxalate)_2_ complex is expected to behave similarly to Ti(Citrate)_3_ in solution. Monotitanyl oxalate species are stable in the pH 1.0 to pH 4.0 range [34]. At higher pH values, multimeric oxoTi(IV) oxalate species are likely present (tetrameric species have been crystallized) [54,55,56] and are labile to ligand exchange, able to form mixed ligand species [34,52] and serve as precursors for other Ti(IV) complexes [36,57]. The complexes Ti(Deferasirox)_2_, Ti(BHPT)_2_ Ti(naphthalene-2,3-diolate)_3_, and Ti(HBED) are highly stable in solution as monoTi(IV) species over a broad pH range (at least pH 4 to 8) and ligand exchange inert [36,38,48,49,58]. Ti(Deferasirox)_2_, Ti(BHPT)_2_ Ti(naphthalene-2,3-diolate)_3_ are dianionic at pH 7.4 whereas Ti(HBED) undergoes hydrolysis-induced partial ligand dissociation forming the anionic titanyl species TiO(H^+^-HBED)^−^ [36,38,48,49,58].

Prior to this study, the Ti(IV) salicylate complexation had not been extensively characterized. An orange-yellow Ti(salicylate)_3_^2−^ complex readily forms at pH 4.5 from a 100 mM aqueous solution, which had been studied by Dey et al. via single-crystal X-ray diffraction, displaying the three salicylates coordinating the Ti(IV) in a bidentate manner through the carboxylic and phenolic oxygens in an octahedral arrangement [39]. The carboxylic and phenolic oxygens are coordinated in a facial orientation with respect to each other. A similar structure was obtained from a DMF solution [59], but the carboxylic and phenolic oxygens are coordinated in a meridional orientation with respect to each other. However, from a 1-butanol solution, a Ti(IV) trisalicylate compound was crystallized in which one salicylate was bound through the carboxylate oxygen, and two salicylates were bound through the carbonyl oxygen; all three salicylates retained phenolic oxygen coordination [60]. Given our interest in the aqueous speciation of Ti(IV) salicylate, we repeated the synthesis of Dey et al. [39]. Orange block-like crystals were obtained through slow evaporation from pure ethanol. The crystals were thoroughly rinsed using cold ethanol and cold water and analyzed through PXRD. The experimental diffractogram for the material was compared to the simulated diffractogram from the crystallographic information file (CIF) for Dey et al.’s Ti(salicylate)_3_ structure downloaded from the Cambridge Structural Database (CSD) (database identifier: TAQPUX, deposition number: 282262) (Figure S1). The appearance of the same reflections when comparing the diffractograms of one to another suggested that the correct material was produced. EDS and elemental analysis were performed to verify the chemical composition of the compound. Elemental analysis provided a molecular formula of Na_2_[Ti(Salicylate)_3_]·4H_2_O·0.5C_2_H_6_O with a molecular weight of 597.26 g/mol: found(theoretical) C, 44.24(44.04); H, 3.88(3.91). The EDS spectra of the compound exhibit the characteristic signals of the respective metal (Ti(IV)) and other elements (carbon and oxygen atoms), which are present in the molecular structure of salicylate. The elements identified were Ti, C, O, and Na (Figure S2). FT-IR spectra were collected of Ti(salicylate)_3_ and metal-free salicylic acid. Noticeable differences were observed between the two, particularly the reduction in the frequency of the C=O vibrational band of salicylic acid from 1650 cm^−1^ to 1614 cm^−1^ due to Ti(IV) coordination (Figure S3).

**Figure 3:**
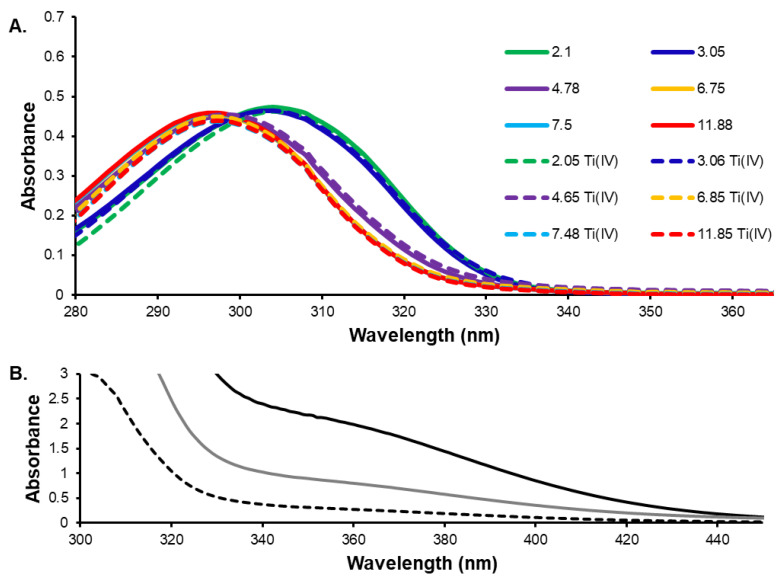
UV-Vis absorbance spectra. (**A**). pH titration of salicylic acid (solid lines) and Ti(IV)-salicylic acid system (dash lines) recorded at different pH values: [ligand] = 150 μM, metal ligand ratio 1:3; *t* = 25.0 °C, I = 0.10 M (NaCl) in 50% (*w*/*w*) ethanol/water]. (**B**). Concentration dependent study of solutions of Na_2_[Ti(Salicylate)_3_] at 250 μM (dashed line), 500 μM (gray line), and 1000 μM (black line) (50:50 DMF:water at pH 4.65 and I = 0.1 M).

The pH-dependent Ti(IV) salicylate complexation was studied by UV-Vis absorbance spectroscopy. Figure 3A demonstrate the spectra of the Ti(IV)-salicylic acid system compared to salicylic acid alone. As can be noted, the spectra are highly similar, and there is no formation of a ligand to metal charge transfer (LMCT) band. Gigant et al. observed an LMCT absorbance between 330–430 nm for their Ti(IV) trisalicylate compound in CCl_4_ solution. This absorbance would be expected of an orange-yellow colored compound and its absence in aqueous solution throughout the pH 2 to 12 range at 50 μM Ti(IV) and 150 μM salicylate indicates a high water exchange labile Ti(IV) complexation by salicylate at micromolar concentration, which appears to provide sufficient Ti(IV) binding to prevent hydrolysis induced precipitation. This water exchange lability is comparable to what is observed for Ti(IV) complexation by citrate [50,51,52,53]. Na_2_[Ti(Salicylate)_3_] demonstrated an effect on MRC-5 cell viability distinct from free salicylate and even the highly unstable titanocene dichloride supports that some form of Ti(IV) binding is present. This solution behavior is very different from what is exhibited by the catecholate chelation of Ti(IV) [17]. In aqueous solution at pH 7.4 and micromolar concentration, a pronounced LMCT is observed for the Ti(IV) chelation by the catecholate moiety (ε_365nm_ = 40,000 M^−1^cm^−1^ for [Ti(naphthalene-2,3-diolate)_3_]^2−^; ε_370nm_ = 9300 M^−1^cm^−1^ for [Ti(catecholate)_3_]^2−^) [38,61]. Thus the wavelength shift observed in the titration from low pH to high pH in the 50% (*w*/*w*) ethanol/water solution is owed to the deprotonation of the carboxylic acid moiety of the salicylic acid (Figure 3A). The pKa of the carboxylic acid in 50% (*w*/*w*) ethanol/water is pKa 3.47 [62], and of the phenolic group is 13.61 in water [63]. A concentration-dependent study of solubilized Na_2_[Ti(Salicylate)_3_] (50:50 DMF:water at pH 4.65 and I = 0.1 M ) was monitored by UV-Vis absorbance spectroscopy to identify a concentration at which the putative Ti(IV) trisalicylate solution species is stable (Figure 3B). A clearly defined LMCT shoulder at 360 nm could be identified at 1 mM concentration with an extinction coefficient of 1900 M^−1^cm^−1^. Dissolving Na_2_[Ti(Salicylate)_3_] directly into pH 7.4 buffer produces a colorless solution, which suggests that although the compound is far more water soluble at this pH, the salicylate is far more ligand exchange labile.

### 3.3. CO-ADD Screening Results

The CO-ADD performs a first antimicrobial screening of compounds at a one-dose concentration of 32 μg/mL to assess general potency. Compounds that demonstrate inhibition of the growth of two or more of the microbes between 50% to 75% or of at least one microbe at greater than 75% are typically then advanced to a dose–response screening (0.25 to 32 μg/mL) and are also tested for human cell toxicity to evaluate their therapeutic index (selectivity against microbes versus human cells). Table 2 provide a summary of these two screenings, which lists the compounds numerically as specified in Figure 1. The compounds Ti(Deferasirox)_2_ (**10**), Ti(HBED) (**11**), K_2_[Ti(naphthalene-2,3-diolate)_3_] (**5**), K_2_[TiO(Oxalate)_2_] (**14**), and HBED (**8**) met the criteria to advance to the second screening. Although deferasirox (**4**) and [Cu(Deferasirox)(H_2_O)]_2_ (**15**) did not meet the criteria, they were also included in the second screening.

Only two compounds demonstrated any note-worthy antimicrobial activity. K_2_[TiO(Oxalate)_2_] (**14**) exhibits a MIC of 32 μg/mL and maximum % inhibition (D_max_) of 77.3% against *C. albicans*. Ti(Deferasirox)_2_ (**10**) exhibits an MIC of 16 μg/mL and maximum % inhibition (D_max_) of 98.2% against *S. aureus* (MRSA). At all concentrations examined, both compounds showed insignificant antiproliferative effect against the HEK293 and red blood cell lines, and thus CC_50_, HC_10_, and HC_50_ values could not be measured. The other five compounds included in the second screening were inactive toward inducing hemolysis. Ti(HBED), K_2_[Ti(naphthalene-2,3-diolate)_3_] (**5**), and deferasirox (**4**) were also inactive in inhibiting HEK293 proliferation. However, HBED (**8**) and [Cu(Deferasirox)(H_2_O)]_2_ (**15**) inhibited the proliferation of the cells at nearly 50% at the maximum concentration tested.

## 4. Discussion

Although the CO-ADD screening did not produce many positive hits, assessing the results has been very insightful about the importance of the Ti(IV) compound identity to the antimicrobial behavior observed or lack thereof. Previous studies centered on soluble Ti(IV) compounds have pointed to a potent antibiotic behavior of the titanyl species with and without bound sulfate at low pH that could be owed to bacterial serine protease inhibition [27,32,33,35]. Our library of Ti(IV) compounds was screened at pH ~7.4, and at this pH, they vary in terms of their solution stability and ligand exchange lability. The highly unstable titanocene dichloride, which rapidly dissociates and precipitates hydrolyzed Ti(IV), demonstrated virtually no promising antimicrobial behavior. The ligand labile compounds K_2_[Ti(Citrate)_3_] and Na_2_[Ti(Salicylate)_3_] were also not promising whereas K_2_[TiO(Oxalate)_2_] demonstrated modest behavior. It could inhibit *C. albicans* proliferation at greater than 75% and exhibited no toxic behavior against the three human cell lines (MRC-5, HEK293, and red blood cells) tested, thus indicating that it displays a significant antifungal therapeutic index. When considering its *C. albicans* MIC value in units of molarity (90.4 μM) (Table S1), this concentration is relatively high. The absence of antibiotic activity of this compound is consistent with the observations of McCue et al. [27,33,35], in which antibiotic activity could only be observed at low pH (≤pH 1), in which the compound does not remain intact and presumably the activity is due to oxalate-free titanyl [33,35]. The lack of antibiotic behavior of Na_2_[Ti(Salicylate)_3_] is disappointing given the activity observed by Julius Pick of Ti(IV) monosalicylate and disalicylate in treating tuberculosis, presumably due to the inhibition of *Mycobacterium tuberculosis* [27]. There are a few unknowns given that we did not work with the same compound formulation and did not screen against *Mycobacterium tuberculosis*. In addition, the conditions of Pick’s work may have produced a different Ti(IV) speciation especially given the topical and oral administration of the compounds [27]. Interestingly, Ti(IV) salicylate (composition not known to us) is used as a preservative in cosmetics products, although experts have already established insufficient data to determine the compound’s safety or true efficacy [64].

Of the solution stable and ligand exchange inert compounds, Ti(Deferasirox)_2_ was the only compound to produce a positive hit, effectively inhibiting the proliferation of MRSA at nearly 100% at a MIC of 16 μg/mL or 20.2 μM. Ti has been previously studied as a possible anti-MRSA agent. Kikushi et al. studied pure Ti against MRSA, but the metal alone did not show significant activity [65]. There are various studies that demonstrate TiO_2_ NPs as possible MRSA inhibitors [66,67,68]. In combination with established antibiotics, the NPs are effective in reducing the MRSA resistance against the antibiotics [66,68]. Recently Ajsuvakova et al. evaluated the dose–response effect of Ti(citrate)_3_^8−^ on hemolytic and nonhemolytic *S. aureus* cultures [53]. They found significant inhibition of their growth rate in only the early phase of the bacterial growth cycle but only at the very high concentration of 50 mM, which would likely be toxic to human cells. However, at all concentrations tested (0.5 to 50 mM) Ti(citrate)_3_^8−^ significantly activated biofilm formation, an undesired result [53]. The Ti(Deferasirox)_2_ MIC value against MRSA is thus a promising result especially given that at this concentration, the compound displays insignificant toxicity against HEK293 and red blood cells and moderate toxicity against MRC-5 cells [36], which suggests a favorable antibacterial therapeutic index.

Ti(Deferasirox)_2_ was designed by us as an anticancer agent who operates intracellularly by transmetalation with the labile iron pool to inhibit Fe bioavailability and enable Ti(IV) release. We have recently proposed that the compound, through its Fe binding and Ti(IV)-induced phosphate hydrolysis of nucleotides, may serve as a dual inhibitor of the iron-dependent human ribonucleotide reductase (RNR class Ia) enzyme, which plays a key role in DNA replication and repair [36,58]. This enzyme is composed of two homodimeric subunits: α2 and β2. The β2 subunit harbors the essential diferric-tyrosyl radical cofactor that initiates the enzyme’s radical chemistry. The Fe binding property of Ti(Deferasirox)_2_ may play a significant role in its inhibition of MRSA by disrupting Fe bioavailability. This possibility is supported by the recent preprint work by Sessler et al., which demonstrated that a prochelator form of deferasirox could inhibit MRSA up to 70% at 15 μM [4,69]. Ti(Deferasirox)_2_ can be viewed as a prochelator with it displaying high extracellular stability and intracellular Fe-induced transmetalation [36]. Ti(Deferasirox)_2_ may also be able to inhibit the bacterial RNR. *S. aureus* contain class Ib and class III RNRs, which are essential for aerobic and anaerobic growth [70,71]. Class Ib RNR is the most commonly observed class and is similar to the class Ia RNR, being composed of two homodimeric subunits [70]. The behavior of Ti(Deferasirox)_2_ is very clearly a synergism between titanium and the ligand, as observed by comparing its potency with metal-free deferasirox and the copper(II) compound of deferasirox (Cu(Deferasirox)(H_2_O)]_2_). The metal-free ligand exhibits significantly less potency than Ti(Deferasirox)_2_ with a maximal inhibition of 44.31% at 32 μg/mL or 85.7 μM; roughly two times the ligand content than Ti(Deferasirox)_2_ at its MIC value ([deferasirox] = 40.4 μM) and with less than half the activity. The Cu(II) compound of deferasirox [Cu(Deferasirox)(H_2_O)]_2_ is even less potent than either Ti(Deferasirox)_2_ or deferasirox, demonstrating a maximal inhibition of 26.9% at 32 μg/mL or 35.3 μM ([deferasirox] = 70.6 μM). The behavior of [Cu(Deferasirox)(H_2_O)]_2_ is consistent with our finding that the compound in solution is very stable, and the ligand has a much higher affinity for Cu(II) than previously reported, competitive with Fe(III) binding [41]. Fe(III)-induced transmetalation likely does not occur for [Cu(Deferasirox)(H_2_O)]_2,_ and thus the compound would not affect the Fe levels in MRSA. When substituting deferasirox with the analogue BHPT, which lacks the benzoic acid group, the metal-free ligand and the Ti(BHPT)_2_ compound are inactive against MRSA. This could be a consequence of poor cellular uptake for the BHPT ligand. Why Ti(Deferasirox)_2_ does not display activity against the other microbes in the CO-ADD screen could also be an issue of cellular uptake, and thus the study of its mechanism against MRSA is highly warranted.

## 5. Conclusions

Having developed a library of compounds and in light of the global need to combat the decreasing pool of effective antimicrobial compounds because of the aggressive resistance of pathogenic agents, an opportunity was sought to explore the potential for Ti(IV) compounds. After the screening of eight Ti(IV) compounds and some of their corresponding ligands by the CO-ADD, Ti(deferasirox)_2_ emerged as a very promising antibiotic agent against MRSA with a significant therapeutic index. It is speculated that this compound may operate by the synergistic behavior of the metal and iron chelator to decrease the bioavailability of Fe(III) and inhibit the microbe RNR activity. Future studies are needed to better understand the mechanism of this compound for the design of a more potent and selective derivative.

## Figures and Tables

**Table 1:** The IC_50_ values (±standard deviation) for the treatment of metal compounds and some corresponding ligands against MRC-5 cells.

Compound	IC_50_ ± SD (µM)	IC_50_ ± SD (µg/mL)	Ref.
Ti(Deferasirox)_2_ (**10**)	25 ± 1	19.8 ± 0.8	[36]
[Cu(Def)(H_2_O)]_2_ (**15**)	8.40 ± 1.62	7.70 ± 1.47	This work
Deferasirox ^a^ (**4**)	Proliferative Behavior		[36]
Ti(BHPT)_2_ (**9**)	2.4 ^b^	1.5	[36]
BHPT (**3**)	>>100	>>25.3	[36]
Ti(HBED) (**11**)	42.0 ± 4.4	18.5 ± 1.9	[49]
HBED ^a^ (**5**)	>>100	>>44.3	[49]
K_2_[Ti(naphthalene-2,3-diolate)_3_] (**8**)	>>100	>>65.5	This work
2,3-dihydroxynaphthalene (**2**)	>>100	>>16.0	This work
Titanocene Dichloride (**13**)	>>100	>>24.9	This work
K_2_[TiO(Oxalate)_2_] (**14**)	>>100	>>35.4	This work
Na_2_[Ti(Salicylate)_3_] (**12**)	8.60 ± 1.54 ^c^	5.14 ± 0.92	This work
Salicylic Acid ^a^ (**6**)	>>100	>>13.8	This work
K_2_[Ti(Citrate)_3_] (**7**)	>>100	>>73.2	This work
Na_3_Citrate (**1**)	>>100	>>29.4	This work

^a^ Note that at pH 7.4, deferasirox will be monoanionic, HBED will be dianionic, and salicylic acid will be monoanionic. ^b^ An approximated relative IC_50_ value. The compound inhibits cell proliferation at a maximum of 25%. ^c^ A relative IC_50_ value. The compound inhibits cell proliferation at a maximum of 50%.

**Table 2:** Predicted or measured MIC (μg/mL) values for the compounds against the Gram-negative (G−ve) bacteria, Gram-positive (G+ve) bacteria, and the fungi. The maximum % inhibition (D_max_) exhibited by the compounds against each organism is included. MIC values that could be measured are highlighted in yellow. D_max_ values of ≥50% are highlighted in gray. ND = Not determined.

	G+ve		G−ve								Fungi			
	*S. aureus* (MRSA)		*A. baumannii*		*E. coli*		*K. pneumoniae*		*P. aeruginosa*		*C. albicans*		*C. neoformans*	
	MIC	D_max_	MIC	D_max_	MIC	D_max_	MIC	D_max_	MIC	D_max_	MIC	D_max_	MIC	D_max_
**1**	>32	10.59	>32	18	>32	6.14	>32	14.22	>32	10.27	>32	0.28	>32	−8.24
**2**	>32	12.55	>32	31.32	>32	6.86	>32	26.77	>32	14.61	>32	16.32	>32	−9.97
**3**	>32	−2.34	>32	−1.93	>32	−9.07	>32	4.11	>32	10.03	>32	6.23	>32	−7.23
**4**	>32	44.31	>32	18.95	>32	7.72	>32	20.2	>32	5.82	>32	4.31	>32	−25.58
**5**	>32	24.28	>32	68.29	>32	51.65	>32	52.86	>32	69.01	>32	3.5	>32	−17.49
**6**	>32	13.86	>32	17.87	>32	−0.01	>32	17.1	>32	9.76	>32	30.95	>32	−10.66
**7**	>32	38.01	>32	17.94	>32	10.41	>32	15.43	>32	7.99	>32	44.53	>32	−7.88
**8**	>32	52.5	>32	24.76	>32	2.67	>32	22.94	>32	13.43	>32	57.57	>32	−1.45
**9**	>32	7.82	>32	1.55	>32	−0.56	>32	6.35	>32	10.27	>32	4.27	>32	−1.7
**10**	16	98.2	>32	16.28	>32	20.44	>32	34.61	>32	7.57	>32	5.74	>32	−5.72
**11**	>32	61.46	>32	68.73	>32	48.02	>32	56.3	>32	72.48	>32	10.43	>32	−18.86
**12**	>32	3.66	>32	9.4	>32	2.6	>32	12.46	>32	9.57	>32	49.3	>32	−5.2
**13**	>32	51.55	>32	15.78	>32	23.76	>32	16.65	>32	16.01	>32	16.15	>32	6.5
**14**	>32	40.5	>32	8.2	>32	11.0	>32	17.2	>32	7.5	32	77.3	>32	ND
**15**	>32	26.9	>32	7.8	>32	−9.5	>32	7.7	>32	19.2	>32	34.0	>32	ND

## Data Availability

Not applicable.

## Supplementary Materials

The following supporting information can be downloaded at: https://www.mdpi.com/article/10.3390/antibiotics11020158/s1, Figure S1: Powder X-ray diffractogram overlay of the simulated Ti(salicylate)_3_ obtained from the Cambridge Structural Database deposition number 282262 (bottom, blue) and synthesized Ti(salicylate)_3_ (top, orange), Figure S2: Energy dispersive spectra of Ti(salicylate)_3_ complex displaying the presence of atoms (carbon and oxygen) present in the ligand and the metal (titanium), Figure S3: FTIR spectrum of salicylic acid (bottom, black) and Ti(salicylate)_3_ (top, orange), Table S1: Concentrations of the compounds tested by the CO-ADD converted from μg/mL to micromolar.
