# Supplementary material for: Exploring Titanium(IV) Complexes as Potential Antimicrobial Compounds
Source: Antibiotics (Basel). 2022 Jan 26;11(2):158. doi: 10.3390/antibiotics11020158 (PMC8868518; doi:10.3390/antibiotics11020158)
Supplement: Supplementary file 1 [file antibiotics-11-00158-s001.zip › antibiotics-1561699-supplementary.pdf]

Exploring Titanium(IV) Complexes as Potential Antimicrobial Compounds

Supporting Information

Israel Rodríguez, Lauren Fernández-Vega, Andrea N. Maser-Figueroa, Branlee Sang, Patricia González-Pagán, and Arthur D. Tinoco

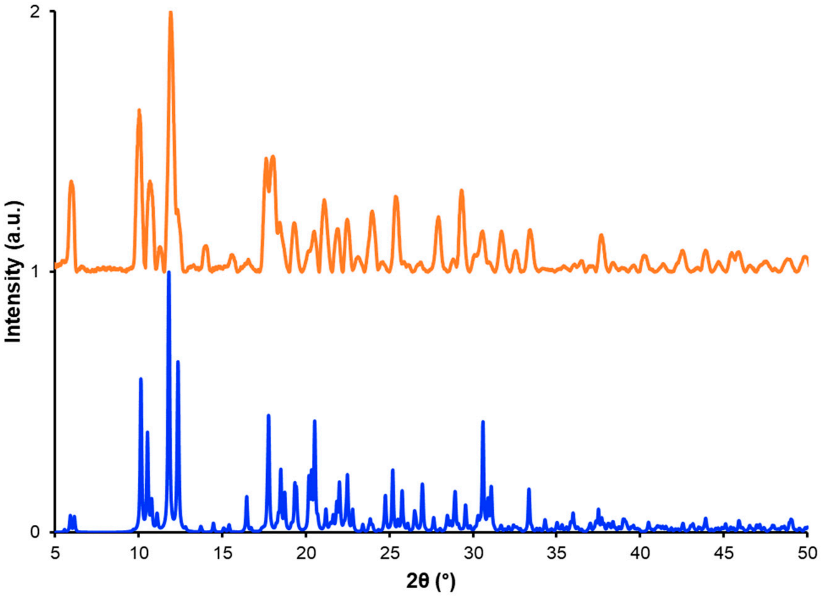

Figure S1. Powder X-ray diffractogram overlay of the simulated Ti(salicylate)$_3$ obtained from the Cambridge Structural Database deposition number 282262 (bottom, blue) and synthesized Ti(salicylate)$_3$ (top, orange).

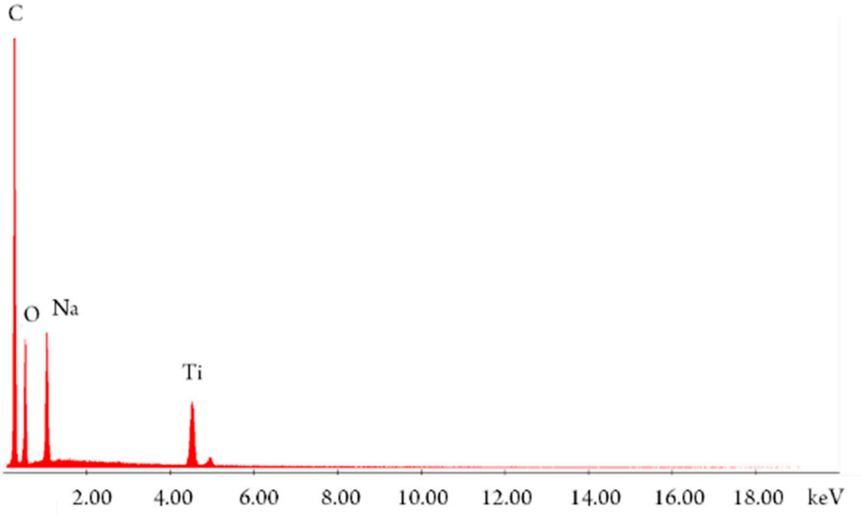

Figure S2. Energy dispersive spectra of Ti(salicylate)$_3$ complex displaying the presence of atoms (carbon and oxygen) present in the ligand and the metal (titanium).

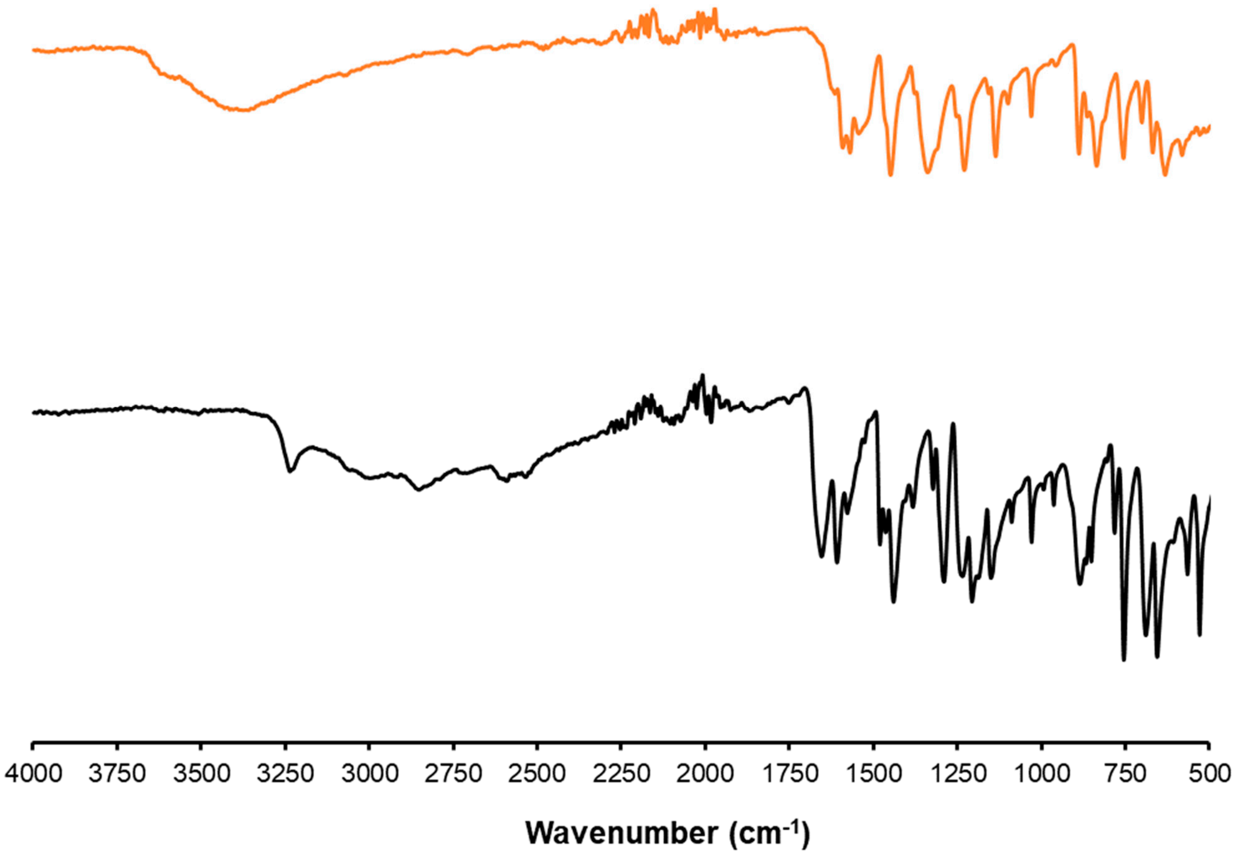

Figure S3. FTIR spectrum of salicylic acid (bottom, black) and Ti(salicylate)$_3$ (top, orange).

Table S1. Concentrations of the compounds tested by the CO-ADD converted from µg/mL to micromolar.

µg/mL	32	16	8	4	2	1	0.5	0.25
**Compound**	Concentration (µM)							
1	108.8	54.4	27.2	13.6	6.8	3.4	1.7	0.9
2	199.8	99.9	49.9	25.0	12.5	6.2	3.1	1.6
3	126.4	63.2	31.6	15.8	7.9	3.9	2.0	1.0
4	85.7	42.9	21.4	10.7	5.4	2.7	1.3	0.7
5	72.3	36.1	18.1	9.0	4.5	2.3	1.1	0.6
6	231.7	115.8	57.9	29.0	14.5	7.2	3.6	1.8
7	43.7	21.8	10.9	5.5	2.7	1.4	0.7	0.3
8	48.9	24.4	12.2	6.1	3.1	1.5	0.8	0.4
9	49.7	24.8	12.4	6.2	3.1	1.6	0.8	0.4
10	40.5	20.2	10.1	5.1	2.5	1.3	0.6	0.3
11	72.5	36.2	18.1	9.1	4.5	2.3	1.1	0.6
12	53.6	26.8	13.4	6.7	3.3	1.7	0.8	0.4
13	128.5	64.3	32.1	16.1	8.0	4.0	2.0	1.0
14	90.4	45.2	22.6	11.3	5.7	2.8	1.4	0.7
15	35.3	17.7	8.8	4.4	2.2	1.1	0.6	0.3
